# Effect of High-Induction Magnetic Stimulation on Elasticity of the Patellar Tendon

**DOI:** 10.1155/2018/7172034

**Published:** 2018-08-01

**Authors:** Jaroslav Prucha, Vladimir Socha, Viera Sochova, Lenka Hanakova, Slobodan Stojic

**Affiliations:** ^1^Faculty of Biomedical Engineering, Czech Technical University in Prague, Sitna Sq. 3105, 272 01 Kladno, Czech Republic; ^2^Laboratory of Human Factors and Automation in Aviation, Czech Technical University in Prague, Horska 3, 128 03 Prague, Czech Republic; ^3^Soreha Ltd., Rehabilitation Clinic at Moldava nad Bodvou, Postupimska 27, 040 22 Kosice, Slovakia

## Abstract

Nowadays, a high-induction magnetic stimulation is starting to be increasingly applied as a biophysical stimulation in the conservative treatment of the degenerative locomotor system diseases. These are mainly in correlation with the changes in soft tissue elasticity, which should be positively influenced by the flow-induced electrical currents of high current density during high-induction magnetic stimulation. This assumption was verified within the interventional and prospective study using the ultrasound elastography. The group consisted of 6 volunteers, whose elasticity of the patellar tendons was measured using the 2D shear-wave ultrasound elastography. The volunteers were then exposed to a 20-minute high-induction magnetic stimulation session with a frequency of 20 Hz, in 2 s package intervals, with a 5 s pause, and a induced electric current density of 100 Am^−2^ in the tendons area. A tendon tension was measured five times for all volunteers, where mean tension at the marked area of the tendon, as well as the highest point tension indicated by the Q-Box, was monitored. The measurement results show that high-induction magnetic stimulation has an influence on the patellar tendon tension change, which occurred in the case of all involved volunteers when the patellar tension was decreased.

## 1. Introduction

High-induction magnetic stimulation (HIMS) is starting to be increasingly applied in the context of conservative treatment of degenerative locomotor system diseases. Due to a rapid and accurately defined time change of the high-intensity magnetic field, the bounce of induced electrical voltage and consequently induced electrical current is created in the treated tissues [1, 2].

This type of stimulation is therefore used in the cases where it is necessary to induce the required current in the target tissues to promote action. The intensity of a magnetic field in the HIMS is between 0.1 and 2.5 T in contrary to low-intensity fields (<0.1 T). However, in both approaches, positive results have been recorded for various applications in conservative treatment of degenerative locomotor system diseases (see [3] and references therein).

Using the high-induction magnetic stimulation instruments with the current technical parameters, it is feasible to cause a voltage drop of approximately 0.0006 mV on the nerve cell membrane, which has a thickness of approximately 6 nm or 4 times less than the threshold value of the neuron potential [4]. Therefore, the high-induction magnetic stimulation cannot affect the creation of an action potential in the body of neuronal cells. This is an important and positive factor testifying frugality and safety of this potential physiological therapeutic method.

As previously mentioned, the gradient of induced electrical voltage within the nerve axons is already sufficiently high for creation and propagation of the induced stimulus [5]. When the synapse is reached, another natural spread of the nerve impulse will be ensured, causing the locomotor response [2]. An irritation of the respective nerve receptors and perceptual therapeutic effects can be sensitively induced by the same mechanism.

The vast majority of the available literature on HIMS is not focused on the field of physiotherapy. The application of HIMS is mostly associated with transcranial magnetic stimulation (TMS), so-called repetitive transcranial magnetic stimulation (rTMS). The main focus of these studies are mostly after brain stroke interventions [6], including paralysis and neglect syndrome [7] or other neurological disorders such as depression [8, 9] or epilepsy [10].

It is important to notice that the effects of pulsed magnetic stimulation of various intensities and frequencies already show a beneficial impact during the treatment of degenerative diseases of the locomotor system [11, 12], strokes or central nervous system injuries, muscular misbalance of the axial body system and/or musculoskeletal disorders [13], osteoporosis [14], neuropathy of the limbs [15], or perfusion disorders in treated tissues [16, 17]. Besides its potential analgetic effect [11, 18], abovementioned effects could often be associated with changes in a soft tissue elasticity [19]. These changes in a soft tissue tension could be positively influenced by flow-inductive currents characterised by high current density.

However, the effect of high-induction magnetic stimulation on the change in tension in soft tissues was not a topic of any research. This on the other hand could have a positive impact on better understanding of the effects of this biophysical stimulation in frame of the conservative treatment of degenerative diseases of the locomotory system. Knowing the HIMS properties could lead to a more precise definition of its indications and contraindications, respectively. Due to these reasons, a main purpose of this study was to verify the hypothesis of a tension change in the patellar tendon (ligamentum patellae) after application of HIMS, using 2D shear-wave ultrasound elastography.

## 2. Materials and Methods

### 2.1. Participants

The study was performed as a pilot and preclinical project, in a form of a case-series study, involving 6 healthy volunteers. All subjects were male, aged between 29 and 60 years. The volunteers did not have any prior health complication records of the studied body part, that is, the patellar tendon. The patients' medical history did not show any locomotor system diseases, systemic diseases, or any other diseases that could affect the elasticity of the studied tendon. The medical history did not show any injuries or surgery interventions as well.

The entire study was conducted under medical supervision, in accordance with the Helsinki Declaration [20], with the informed consent of the experiment participants. The study was approved by the local ethical commission (Faculty of Biomedical Engineering, Czech Technical University in Prague).

### 2.2. Measuring Procedure

The experiment itself consisted of two parts: (1) before the HIMS application and (2) after the HIMS application. Prior to the experiment, each volunteer was in a relaxed, low-lying position for 20 minutes with a slight lower limbs flexion. The expected goal here was to eliminate the effect of the previous physical activity on the mechanical properties of the tendon or to prevent spontaneous release of the observed structure during the experiment. After this initiation phase, 2D shear-wave ultrasound elastography was performed [21]. The following ultrasound elastography was performed 20 minutes after the HIMS procedure.

The high-induction magnetic stimulation was performed using the Saluter Moti® (EMBITRON-CertiCon Group, Prague, Czech Republic). The use of this device allows selection of the intensity, frequency, and a shape of the pulse packages. A used applicator is designed to treat larger tissue volumes. The device was set in order to reach a perceptive and finely motoric effect, according to the parameters stated by the manufacturer (frequency 20 Hz, 2 s package length, 5 s pause, magnetic induction of 2.5 T, and induced electric current density in the tendon area 100 Am^−2^). The length of the procedure was set to be 20 minutes.

**Figure 1:**
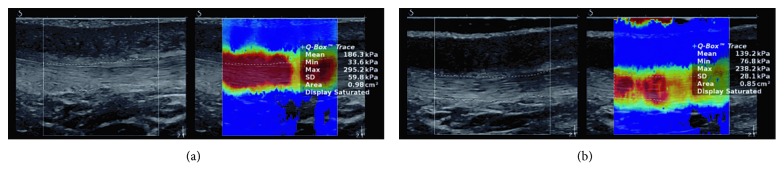
Quantitative evaluation of shear stress in the patella tendon using area evaluation of ultrasound images before (a) and after (b) the high-induction magnetic stimulation.

**Figure 2:**
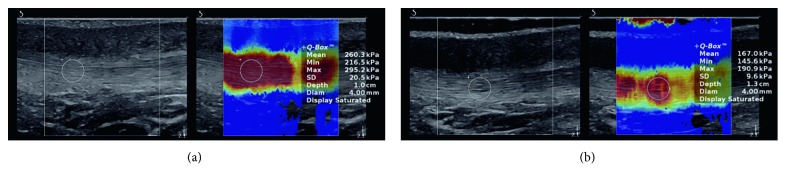
Quantitative evaluation of shear stress in the patella tendon using most saturated point of ultrasound images before (a) and after (b) the high-induction magnetic stimulation.

In order to keep the uniformity of the measurements, the volunteer's position in both cases (before and after HIMS application) was the same [22]. The volunteers layed relaxed on their back, with slightly flexed lower limbs (10°–15° defined by goniometric measurement) and with the hands along the body. For measuring elasticity of the patellar tendon, real-time ShearWave™ elastography was utilized, using the ultrasound Aixplorer® (SuperSonic Imagine, Aix-en-Provence, France). The measurement was performed in the SWE setting in order to scan in the grayscale B-mode with a real-time color elasticity map superimposed (Figures 1 and 2), with “knee” tissue preset setting using a linear probe SL10-2 (2–10 MHz), according to manufacturer recommendation [23]. The patellar tendon was measured in the middle point, between the apex patellae and the contact spot of a tendon at the tuberositas tibiae. The measurement location was determined mainly due to a simple identification of the tendon course and simpler elimination of the influence of other tissue structures.

In order to eliminate the variability of the individual measurements, at least five elastographic image recordings were performed for each volunteer, before and after HIMS applications. Such variability of measurement may be caused by uneven localization of the considered segment, the pressure applied on the probe, or the use of an insufficient amount of coupling gel [24].

### 2.3. Data Processing and Statistical Analysis

The patellar tendon elasticity values were obtained through a post hoc image processing, in order to prevent an influence of on-site evaluation on ultrasound elastography investigation. The values of the elasticity were obtained by applying the two methods, by defining the visualized area of the tendon (Figure 1), and by evaluating the most saturated point with Q-Box, having a 3-4 mm diameter (Figure 2). The median of shear stress from the recorded image was determined to be a quantitative value for data sets creation, as recommended by Kot et al. [24].

The data for further processing were divided into two basic groups according to the quantitative evaluation method used, that is, the area boundaries setting (M1) and the most saturated point (M2).

Before the overall (pretest-posttest) evaluation, the calculation of the average value for each volunteer was performed. As previously mentioned, the data for each volunteer contained at least 5 repeated measurements in order to assess variability in the measurement itself. Even though these measurements appear to be reliant, it should be noticed that individual measurements before and after the procedure do not present pairs. These data were not measured exactly at the same point on the tendon, and the series of measurements were not possible to be performed before and after the procedure exactly on the same point, which are not pairwise dependent (i.e., with order retention). Therefore, these results are presented individually in the form of boxplots, characterizing the distribution of the observed tendon tensions before and after the application of the HIMS for each participant separately. For an orientation assessment whether the data of individual volunteers before and after the HIMS originated from the distribution with the same median, the Wilcoxon rank-sum test [25] was performed at the level of significance *α*=5%. The statistically significant difference between the mean values of the tendon tension was then defined for *p* < 0.05.

To create a group distribution of characteristic values of the measured patellar tendon tensions before and after the procedure, the medians of the previously mentioned 5 measurements were used, separately for each subject. Since the data collection process and previous processing indicates that individual data sets (M1 and M2) contain data before and after the procedure, it could be claimed that there is a mutual dependence. It could also be assumed that the data do not have normal distribution. Therefore, a nonparametric Wilcoxon signed-rank test was used to evaluate the statistical significance of the change between the tendon tension before and after the procedure within the monitored group of participants [25]. The test is used to verify the hypothesis that, for two related samples, matched samples or repeated measurements on a single sample population mean rank differ. The significance level for the zero hypothesis testing (that the pair data originate from a zero median difference distribution) was set to *α*=5%.

Statistical analysis was performed in Matlab environment using built-in statistical toolbox (Matlab R2015b, MathWorks, Inc., Natick, MA, USA).

## 3. Results

Using Wilcoxon's rank-sum test during evaluation of the statistical significance of median differences, before and after the HIMS, it was found among individual volunteers that there is a statistically significant difference before and after the application of HIMS among all volunteers. The *p* value of the test statistics was in all cases lower than 0.05 in both monitored groups dependent on the evaluation methods M1 and M2.

**Figure 3:**
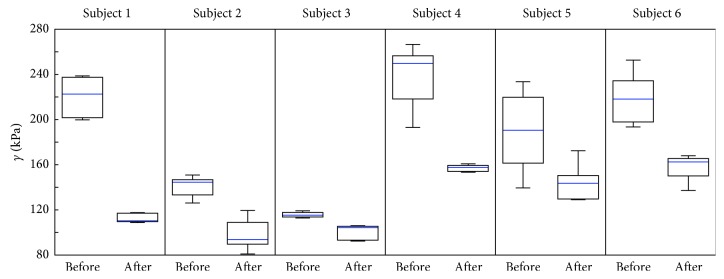
Results of measured shear-stress (*γ*) distribution gathered through area boundary setting method (M1), by evaluation of ultrasound images before and after the application of HIMS for particular subjects.

The results of the tendon tension evaluation for individual volunteers using method M1 are presented in Figure 3 in the boxplots form. In the case of Subject 1, recorded median tension drop was from 222.6 kPa to 110.3 kPa, and the test statistic had *p*=0.0022. In case of Subject 2, recorded median tension drop was from 176.8 kPa to 100.7 kPa, and the test statistic had *p*=0.0079. In case of Subject 3, recorded median tension drop was from 115.3 kPa to 104.4 kPa, and the test statistic had *p*=0.0047. In the case of Subject 4, recorded median tension drop was from 243.5 kPa to 133.1 kPa, and the test statistic had *p*=0.0010. In case of Subject 5, recorded median tension drop was from 195.0 kPa to 150.4 kPa, and the test statistic had *p*=0.0480. In case of Subject 6, recorded median tension drop was from 219.5 kPa to 147.25 kPa, and the test statistic had *p*=0.0001.

**Figure 4:**
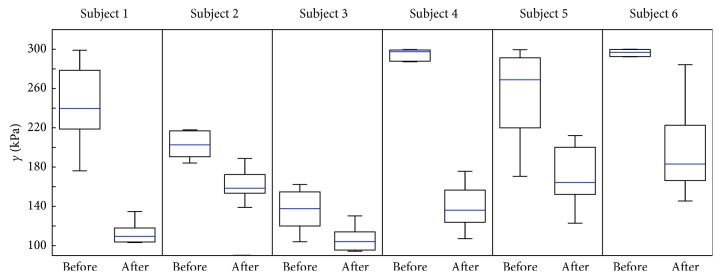
Results of measured shear-stress (*γ*) distribution gathered through evaluation of the most saturated point of ultrasound images (M2) before and after the application of HIMS for particular subjects.

The results of the tendon tension evaluation for particular volunteers using method M1 are presented in Figure 4. In case of Subject 1, recorded median tension drop was from 239.9 kPa to 109.3 kPa, and the test statistic had *p*=0.0022. In case of Subject 2, recorded median tension drop was from 202.6 kPa to 158.4 kPa, and the test statistic had *p*=0.0159. In case of Subject 3, recorded median tension drop was from 137.6 kPa to 104.1 kPa, and the test statistic had *p*=0.0221. In case of Subject 4, recorded median tension drop was from 297.5 kPa to 136.1 kPa, and the test statistic had *p*=0.0010. In case of Subject 5, recorded median tension drop was from 268.8 kPa to 164.2 kPa, and the test statistic had *p*=0.0033. In case of Subject 6, recorded median tension drop was from 296.9 kPa to 183.15 kPa, and the test statistic had *p*=0.0001.

The results show that the median of distributions measured before the HIMS application is statistically significantly higher than the median derived from the distribution of the values measured after the HIMS application for all subjects. This also applies for both processing methods, that is, M1 and M2.

By using the presented results, it is possible to determine with a high degree of certainty the reference values of the tension on the patellar tendon, around which the other measured values oscillate. The medians of the individual distributions presented in Figures 3 and 4, which determine the central value of the presented distributions, were used to create complex data sets for M1 and M2.

**Figure 5:**
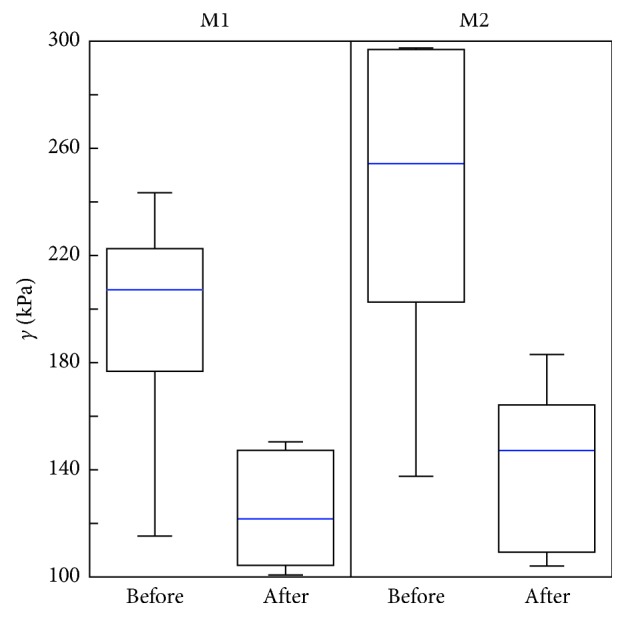
Integrated results of measured shear-stress (*γ*) distribution for group of subjects, gathered by the area boundary setting (M1) and the most saturated point (M2) method before and after the HIMS application.

In Figure 5, the distributions of tendon tensions for M1 and M2 are presented. In the case of M1, the median tension dropped from 207.3 kPa to 121.7 kPa, and the test statistic had *p*=0.0313. In the case of the M2 method, the median tension dropped from 254.3 kPa to 147.3 kPa, and the test statistic had *p*=0.0313. Pairwise analysis performed using the Wilcoxon signed-rank (paired) test showed that, in both cases, M1 and M2, there was a significant decrease in the median value of the tendon tension prior to the application of the HIMS in comparison with the measurements performed after the procedure. It means that sufficient amount of evidences were not found in order to confirm the hypothesis that the two dependent measurement samples originate from the distribution with median difference equal to 0, at the significance level *α*=0.05 (in both cases, it is *p* < 0.05). These findings confirm the previous analysis.

## 4. Discussion

From the results presented in the form of boxplots in Figures 3 and 4 and with the *p* values presented in Results, it is visible that, in the case of all volunteers, a statistically significant change in tendon tension after the HIMS application occurred. Moreover, as it is apparent from the results presented in the boxplots in Figure 5 and from the *p* values presented in Results, in the cases of both methods, there was a statistically significant decrease of the median tension comparing the measurements before and after the application of HIMS. Therefore, it could be stated that both methods are suitable for obtaining the tension values that could be subsequently used for data analysis purposes.

The boxplots also show an interindividual variability in the monitored group of healthy participants. It is possible that this intervariability is caused by a different level of day-to-day activity among the studied subjects, physiological predispositions, and of course, age. Ozcan et al. [26] in their studies did not find statistically significant differences in the patellar tendon tension comparing the professional athletes and healthy volunteers, and therefore, it is not confirmed that day-to-day activities such as sports or different levels of physical activity affect elasticity in a healthy population. On the contrary, Hsiao et al. [27] found that there is a reduction in the elasticity of the patellar tendon within the monitored group of older individuals aged 60−70 years (176.2 ± 45.9 kPa) in comparison to the group of subjects aged 20−30 years (243.1±61.4 kPa) and the group of subjects aged 40–50 years (238.7±73.7 kPa).

In various studies, the authors also observed relatively high intervariability of the measurements. In addition to the previously mentioned results presented in the study by Hsiao et al. [27], similar intervariability may be observed in the case of a study by Hardy et al. [28] (see standard deviations), where mean shear stress was 50.9±33.1 kPa in case of knee extension, 137.5±50.7 kPa in 30° flexion, and 226.5±60.3 kPa in 90° flexion. Unfortunately, there is currently no study evaluating the factors causing intervariability in tendons shear stress. These factors, however, certainly exist, as is shown by the results of other studies. It is therefore possible that these factors may also affect the effectiveness of HIMS because in the present study it is clear that the subjects response to HIMS are different.

Even though the magnitude of the changes was different among volunteers, there was a significant decrease in tendon tension after the application of HIMS, compared to the tensions measured before the therapy, in all cases. Moreover, the values characterizing shear stress on patellar tendon are comparable with results presented in previous and further mentioned studies.

By presenting the results of the present study in the context of the possible application of HIMS as a biophysical stimulation in the conservative treatment of degenerative diseases of the locomotory system, it should be mentioned that the tendon (and also the ligamentum) consists of a bundle of parallel collagen fibers having relatively low elasticity. The maximum extension of these structures may be about 10% of its length [29]. The course of collagen fibers is partially pointed, making the pull soft and flexible, and ligaments also contribute to a slight and flexible movement in the joint itself. These structures are, however, provided with pain receptors, so that these soft tissues are a frequent source of pain in the locomotory system diseases of various etiopathogenesis. In the case of a locomotive system disease, the elastic properties of the tendons (and ligaments) seem to change.

This statement supports, for example, the research by Dirrichs et al. [30], which focuses on three important tendons (tendon patellae, achilles tendon, and tendon epicondylitis humeri), showing significantly lower Yang modulus of elasticity for pathological tendons (values around 50 to 60 kPa), while healthy tendons have Yang's modulus of elasticity around 150 kPa. The authors, among others, believe that measuring stiffness of tendons by ultrasound elastometry could be a good diagnostic method that could predict their tendinopathies in the case of extremely reduced tendon stiffness. Another work by Zhang et al. [31] is dedicated to measuring the elasticity of achilles tendon after its operative repair. The authors find a gradual increase in stiffness of these tendons from the value around 180 kPa to around 280 kPa. On the contrary, in an article by Leong et al. [32], the authors focused on the measurement of stiffness or elasticity of muscular fibers and tendons of the upper trapezium (musculus trapezius upper) in case of healthy athletes and in tendonopathic athlete's rotator cuff. They found significantly lower values and hence better elasticity or less stiffness of measured structures among athletes without difficulties.

Very important in this context is also work by Wu et al. [33]. The authors focused on ligamentum coracohumeral and found that, in symptomatic patients, the Yang modulus of ligament elasticity was significantly increased (values around 235 kPa in the neutral position of the limb), while healthy individuals have significantly lower stiffness—greater elasticity of this ligament (value around 185 kPa in the neutral position of the limb).

The hypothesis could be stated that soft tissues that enable the movement in the joints through the connective tissue of the muscle to the tendon and the strengthening in the joint of the bones should have optimal toughness, respectively, Yang's modulus of elasticity. Too low Yang modulus, that is, extremely low stiffness, can cause damage to these structures, while excessive rigidity (high Yang modulus) will be followed by degenerative joint disease. The joint will be excessively “stiffened,” the movement ensured by the muscles becomes obstructed, and as a result of these factors, the existing pain receptor will be excessively irritated, which will further reduce the need for a movement. Symptomatic treatment of these conditions should therefore reduce the excessive stiffness of the ligaments and tendons. The ligament and tendon, due to its helical structure of the longitudinally arranged collagen fibers, can increase their elasticity (reduce stiffness, i.e., reducing the Yang modulus of elasticity) by appropriate mechanical exercise—stretching and shortening provided by the muscle movement. However, in a regular rehabilitation exercise, a large number of contractions and loosening should be repeated. It would also be very important to eliminate the risks of excessive stretching by muscle contraction. To do both in rehabilitation exercises is very difficult. high-induction magnetic stimulation provides fine motor effects. The muscles of the stimulated areas gently contract, and their contractions are exercised by the tendons and ligaments around the joint. In our experiment, over 20 minutes of high-induction magnetic stimulation to almost 200 traceable evoked muscle contractions of wavy character, involving up to 7,000 fine motor reactions of muscles occurred. These motile stimuli apparently do not change the proportion of elastin and collagen in the tendon and ligament, but the mechanically stimulated tendon may modify its structure in terms of the mechanical release of the corrugated-to-spiral-forming bundles of collagen fibers. This release is intended to be released in the amorphous intercellular mass.

This hypothetical mechanism is certainly activated by natural movement and targeted rehabilitation exercises. However, with the use of high-induction magnetic stimulation, significant effects are achieved during the short duration of the procedure, resulting in increased elasticity of ligaments and tendons. This increase in elasticity (reduction in stiffness, i.e., reduction in the Yang modulus of elasticity) can not, in principle, exceed the limit of the tendon or ligament threat by its lack of stiffness, as it apparently does not have the ability to affect the ligament or tendon itself. For these reasons, the chance of high-induction magnetic stimulation reducing the excessive stiffness of tendons or ligaments appears, especially in degenerative joint diseases, thereby reducing undesirable symptoms of these diseases and contributing to the possibility of natural movement.

Other effects of high-induction magnetic stimulation applied at the cellular and molecular level cannot be ruled out. Some works, for example, by Prucha et al. [34] indicate the possibility of the beneficial influence of time-varying induced electrical currents during the cell response to inflammatory mediators, or blocking the transmission of information about the pain from the TRPA1 ion channels.

## 5. Conclusion

The study was focused on the application of a high-induction magnetic stimulation as biophysical stimulation and its effect on patellar tendon tension. The individual tension values were obtained through the processing of the images obtained by ultrasonic elastography. The processing was carried out by two methods: M1 (boundaries setting for visualized area of the tendon) and M2 (evaluation of the most saturated point in the image with Q-Box of the 3-4 mm diameter). It is clear from the obtained results that regardless of the chosen method for tendon tension data obtaining, there was a significant decrease in the tension after the application of HIMS. However, this claim cannot be applied generally due to a low number of tested subjects and due to a fact that only one specific tendon was measured (i.e., ligamentum patellae). In particular, the low number of subjects tested is the limitation of this study, so it can be assumed that the research sample does not represent the entire population. Another limitation is a fact that experiment was performed only on the completely healthy volunteers. Anyway, a presence of statistically significant decrease recorded for all subjects indicates the effect of HIMS on the tendon tension. It can be assumed that this method reduces the tendon tension also in the cases of other tendons in the body. In order to reach a high level of statement validity, it is necessary to ensure measurement of a statistically significant set.

This study can therefore be used as a basis for follow-up research focused on the application of HIMS as biophysical stimulation in the conservative treatment of the degenerative locomotor system diseases. If the measurement confirms that, by applying the HIMS method, the tendon tension actually decreases, and it would be beneficial to find out to what extent the motion in the joint is improved after the application of this method (if the extent of joint movement is caused by tendon contracture or shortening after injuries, after surgery, after immobilization, etc.), however, with the subsequent use of manual rehabilitation techniques under the guidance of a physiotherapist. This way, it could be determined to what extent the HIMS method later on facilitates the manual physiotherapist's rehabilitation process.

Another research topic could be application of this method in children rehabilitation. The main benefit could be noticeable in the treatment of children with cerebral palsy, where due to central nervous system involvement, the muscular spasticity, shortening and contracture of tendon, joint deformities, and a reduction in the range of movements occur in varying levels and intensities.

This method could be used mainly in case of those patients whose disease is not caused by a mental, but mainly locomotor-related disorder, and would be very promising in terms of their life quality improvement.
